# Phenotypic Expansion of *PPP1R12A*-Related Syndrome: A Novel Splicing Variant Associated with Hearing Loss and Inner Ear Malformations

**DOI:** 10.3390/genes17080856

**Published:** 2026-07-24

**Authors:** Giulia Pianigiani, Lara Emily Rosso, Anna Morgan, Beatrice Spedicati, Manuela Napoli, Stefano Giuseppe Caraffi, Emanuele Coccia, Valeria Polizzi, Livia Garavelli, Giorgia Girotto

**Affiliations:** 1 Institute for Maternal and Child Health—IRCCS “Burlo Garofolo”, Via dell’Istria 65/1, 34137 Trieste, Italy; anna.morgan@burlo.trieste.it (A.M.); beatrice.spedicati@burlo.trieste.it (B.S.); giorgia.girotto@burlo.trieste.it (G.G.); 2 Department of Medicine, Surgery and Health Sciences, University of Trieste, Strada di Fiume 447, 34149 Trieste, Italy; laraemily.rosso@burlo.trieste.it; 3 Neuroradiology Unit, Azienda USL-IRCCS di Reggio Emilia, Viale Risorgimento 80, 42123 Reggio Emilia, Italy; napoli.manuela@gmail.com; 4 Radiology Unit, Salus Hospital, GVM Care & Research, Via Ulderico Levi 7, 42123 Reggio Emilia, Italy; 5 Medical Genetics Unit, Azienda USL-IRCCS di Reggio Emilia, Viale Risorgimento 80, 42123 Reggio Emilia, Italy; stefanogiuseppe.caraffi@ausl.re.it (S.G.C.); emanuele.coccia@studio.unibo.it (E.C.); livia.garavelli@ausl.re.it (L.G.); 6 Department of Medical and Surgical Science (DIMEC), Alma Mater Studiorum University of Bologna, Via Massarenti 9, 40138 Bologna, Italy; 7 Department of Audiology, Azienda USL-IRCCS di Reggio Emilia, Viale Risorgimento 80, 42123 Reggio Emilia, Italy; valeria.polizzi@ausl.re.it

**Keywords:** *PPP1R12A*, GUBS syndrome, sensorineural hearing loss, inner ear malformations, whole-exome sequencing, phenotypic expansion

## Abstract

**Background**: Pathogenic variants in the *PPP1R12A* gene have been associated with a malformation syndrome involving the brain and the genitourinary systems (GUBS, MIM #618820). To date, neither hearing loss (HL) nor inner ear malformations have been reported in affected individuals, and these features are therefore not currently regarded as part of the *PPP1R12A*-related phenotype. Moreover, functional evidence supporting the pathogenicity of several reported variants remains limited. **Methods**: We investigated a 12.5-year-old patient presenting with profound bilateral sensorineural hearing loss associated with inner ear malformations, genitourinary and central nervous system abnormalities. The patient underwent comprehensive clinical, audiological and radiological assessments, followed by genetic testing via trio-based whole-exome sequencing (WES). The molecular consequences of the identified variant were evaluated through minigene splicing assay and RT–PCR analysis on RNA extracted from peripheral blood cells. **Results**: WES identified a novel heterozygous splicing variant (c.792+3A>C) in the *PPP1R12A* gene (NM_002480.3). Functional studies demonstrated that this variant causes complete skipping of exon 5, resulting in a frameshift and the introduction of a premature termination codon. RT–PCR analysis confirmed the presence of the alternatively spliced transcript lacking exon 5. In addition, an extensive review of the literature indicated that no clear genotype–phenotype correlation has yet been established for *PPP1R12A*-related disorders and, whereas the majority of previously reported patients share brain and genitourinary malformations our patient additionally presented with profound bilateral sensorineural HL and inner ear malformations. **Conclusions**: Our findings suggest that *PPP1R12A*-related disorders may exhibit a broader phenotypic variability than previously recognized and we further propose that HL and inner ear malformations may represent novel features associated with this clinical spectrum. Moreover, our study underscores the importance of functional studies for accurately defining the molecular consequences of novel variants and establishing appropriate clinical correlations.

## 1. Introduction

The Genitourinary and/or Brain malformation Syndrome (GUBS, MIM #618820) is a rare autosomal dominant disorder characterized by a broad spectrum of congenital anomalies mainly involving the urogenital and brain systems. The clinical presentation is highly variable, ranging from minor anomalies that may require only clinical observation, to severe defects requiring intensive medical treatment [1].

The *PPP1R12A* gene (NM_002480.3), also known as *protein phosphatase 1 regulatory subunit 12A* gene (MIM *602021), is located on the long arm of chromosome 12 (12q21.2-q21.3) and spans a region of approximately 162 kb. It comprises 25 exons and undergoes alternative splicing, producing different isoforms of the encoded protein. *PPP1R12A* encodes the myosin light chain phosphatase target subunit 1 (MYPT1) protein, which regulates the catalytic subunit of the protein phosphatase 1 (PP1c) and, together with it and a small subunit of unclear function (M20/M21), forms the myosin phosphatase complex. As a RhoA/ROCK target, MYPT1 is phosphorylated by ROCK, which inhibits myosin phosphatase and raises non-muscle myosin II phosphorylation and actomyosin contractility [2]. This complex plays a key role in regulating myosin light chain phosphorylation, thereby modulating smooth muscle contraction and various cellular processes, including cell cycle, development, gene expression regulation and neurotransmitter release [3]. Also, the RhoA/ROCK pathway plays a fundamental role in cochlear hair cell development by regulating actin polymerization, stereocilia morphogenesis, and planar cell polarity [4], placing MYPT1 within a signaling cascade that is critical for inner ear morphogenesis. Consistent with these observations, mouse transcriptomic datasets available through the gEAR datasets show that *Ppp1r12a* is expressed in cochlear and vestibular sensory epithelia and in supporting cells (E16.5, P0, mouse, RNA-seq, cochlear and vestibular sensory epithelium. gEAR Portal. Retrieved Jul 14, 2026) [5,6]. In addition, single-nucleus RNA-seq from the gEAR Human Inner Ear Atlas broadly detects *PPP1R12A* throughout the human fetal (gestational weeks 7.5 and 9.2) and adult inner ear, in particular in hair cells, glia, endothelial cells and periotic mesenchyme [7]. Despite these observations, the role of PPP1R12A in auditory function remains largely unexplored, as no animal model has specifically addressed an auditory phenotype nor specific functional studies has yet been performed.

Pathogenic variants in the *PPP1R12A* gene have been implicated in the etiology of GUBS. To date, 20 different *PPP1R12A* variants have been reported as disease-causing in the Human Gene Mutation Database (HGMD) (https://my.qiagendigitalinsights.com/bbp/view/hgmd/pro/start.php, last accession on 30 March 2026), including 8 nonsense variants, 2 intronic/splicing variants, 9 small insertions/deletions and 1 complex rearrangement [8,9,10,11,12,13]. Moreover, recent studies have broadened the clinical and mutational spectrum of *PPP1R12A*-related disorders. Tian and colleagues recently reported an additional heterozygous de novo splicing variant (c.1551-2A>G) in the *PPP1R12A* gene in twins presenting with GUBS and disorders of sex development (DSD) [14]. Also, emerging evidence from recent case reports has linked de novo heterozygous variants in *PPP1R12A* to gastrointestinal anomalies, further supporting the involvement of the gastrointestinal system as a significant component of the phenotypic spectrum [15,16]. Finally, recent findings suggest that *PPP1R12A* variants may also cause altered EEG patterns or epileptic phenotype [12,17]. Lastly, *PPP1R12A*-related disorder may be considered within a broader group of conditions caused by variants in genes encoding regulatory components of serine/threonine phosphatase complexes. These multi-subunit holoenzymes are essential for controlling substrate specificity, subcellular localization, and fine-tuning of catalytic activity. Increasing evidence indicates that pathogenic variants affecting phosphatase regulatory subunits, including those of PP1 and PP2A complexes, contribute to a growing spectrum of neurodevelopmental and multisystem disorders [18,19].

In this study, we provide a detailed clinical and molecular description of a male patient, previously reported in an Italian cohort of individuals with hearing loss [20], presenting with a profound bilateral sensorineural hearing loss (SNHL) found in association with inner ear malformations, genitourinary and central nervous system (CNS) abnormalities and carrying a novel splicing variant in the *PPP1R12A* gene. Our clinical evidence, supported by extensive functional characterization of the novel variant identified in this study and a review of the literature, suggests that hearing impairment may be an underrecognized feature of *PPP1R12A*-related disorders, highlighting the need to consider auditory evaluations in patients with this diagnosis and suggesting a phenotypic and mutational expansion of GUBS.

## 2. Materials and Methods

### 2.1. Literature Review

A literature review was conducted using PubMed, and articles containing the keywords “PPP1R12A” and “GUBS” were included. All *PPP1R12A* variants reported in the HGMD Professional Database and classified as “likely pathogenic” or “pathogenic”, and clearly associated with GUBS, were collected.

### 2.2. Sample Collection and Ethical Compliance

The patient was enrolled at the Medical Genetics Unit, Azienda USL-IRCCS di Reggio Emilia (Reggio Emilia, Italy). Whole peripheral blood samples were collected from the proband (II-1) and both parents (I-1, I-2) and sent to the Institute for Maternal and Child Health—IRCCS “Burlo Garofolo” (Trieste, Italy) for subsequent analyses. All analyses were performed following the relevant guidelines and regulations. Written informed consent for genetic testing and research purposes was obtained from all individuals or their next of kin. The study was conducted in accordance with the Declaration of Helsinki.

### 2.3. DNA and RNA Extraction and Quantification

Genomic DNAs (gDNAs) of the proband and both parents were extracted from peripheral blood samples using the QIAsymphony SP instrument in combination with the QIAsymphony DNA Midi Kit (Qiagen, Hilden, Germany). For total RNA extraction, 2.5 mL of peripheral blood of the proband was collected into a PAXgene Blood RNA tube (PreAnalytiX, Qiagen BD), and purification of RNA was performed with the PAXgene Blood RNA Kit (PreAnalytiX, Qiagen BD) following the manufacturer’s instructions. DNA and RNA quality and concentration were assessed using the Nanodrop One spectrophotometer (Thermo Fisher Scientific, Waltham, MA, USA).

### 2.4. Whole-Exome Sequencing

Trio whole-exome sequencing (WES) was carried out at the IRCCS Burlo Garofolo (Trieste, Italy). According to the manufacturer’s instructions, DNA was processed for library enrichment with the Twist Human Core Exome and the Human RefSeq Panel Kit (Twist Bioscience, South San Francisco, CA, USA). Sequencing was performed on a NextSeq 550 platform (Illumina, San Diego, CA, USA). Sequencing quality metrics were as follows: the proband achieved a mean target coverage of 72.7×, with 95.6% of target bases covered at ≥20×; the mother’s sample achieved a mean coverage of 65.1×, with 95.2% of target bases covered at ≥20×; and the father’s sample achieved a mean coverage of 59.8×, with 95.1% of target bases covered at ≥20×. The on-target rate was 28.4% for the proband, 28.9% for the mother, and 29.2% for the father. 

Data processing, including sequence alignment to the human reference genome GRCh37/hg19, variant filtering and prioritization were performed as previously reported [20]. Briefly, sequencing data were processed using the EnGenome Germline Pipeline v2.0. Sequencing reads underwent quality control and adapter trimming using FastQC and TrimGalore, followed by alignment to the human reference genome GRCh37/hg19 using BWA-MEM. PCR duplicates were marked with Picard, and base quality score recalibration was performed according to GATK Best Practices. Single-nucleotide variants (SNVs) and small insertions/deletions (indels) were called using GATK HaplotypeCaller v4.1.2, while exon-level copy number variants (CNVs) were assessed using ExomeDepth. Variants were annotated and interpreted using the eVai platform (EnGenome) and prioritized according to allele frequency in population databases (minor allele frequency < 0.001 in gnomAD), predicted functional impact (SIFT, PaPI score, DANN score, and dbscSNV score), inheritance model, and classification based on the ACMG/AMP guidelines [21].

### 2.5. In Silico Analysis

The potential effect of the variant on pre-mRNA splicing was assessed through in silico analysis by MaxEntScan [22], dbscSNV version 1.1 [23] and the SpliceAI prediction tool version 1.3.1 (https://spliceailookup.broadinstitute.org, accessed on 30 March 2026) [24].

### 2.6. Splicing Minigene Assay

Wild-type and mutant DNA fragments containing *PPP1R12A* exon 5 (145 bp) and around 200 bp of flanking introns were amplified by PCR using the gDNA of the proband as template, the KAPA HiFi HotStart ReadyMix (Roche, Basel, Switzerland) and primers PPP1R12A_XhoI_FW and PPP1R12A_NotI_REV (Table S1). These primers included restriction sites for XhoI and NotI that were used to clone the fragments into the XhoI/NotI-digested Exontrap pET01 vector (MoBiTec GmbH, Göttingen, Germany). Recombinant vectors were verified by Sanger sequencing on a 3500 Dx Genetic Analyzer (Thermo Fisher Scientific), using the Big Dye v3.1 Terminator Sequencing Kit (Thermo Fisher Scientific) and the pET01_EX1_Seq_FW and pET01_EX2_Seq_REV primers (Table S1).

### 2.7. Cell Culture, Transfection and RNA Extraction

Human Embryonic Kidney 293T cells (HEK293T; CRL-3216) were purchased from ATCC and grown in Dulbecco’s modified Eagle medium (DMEM) with L-glutamine (Euroclone, Pero, Milan, Italy) supplemented with 100 U/mL penicillin, 0.1 mg/mL streptomycin (P/S; Euroclone) and 10% fetal bovine serum (FBS; Euroclone). Cells were maintained at 37 °C with 5% CO_2_. Cell line authentication was ensured by the short tandem repeat (STR) profile provided by ATCC (available in the “detailed product information > quality control specifications section” of the ATCC website, https://www.atcc.org/products/crl-3216, accessed on 30 March 2026). Cultures were routinely tested every couple of weeks for the presence of Mycoplasma using the Mycoplasma PCR Detection Kit (Applied Biological Materials Inc. (abm), Richmond, BC, Canada). Mycoplasma-free cells were seeded into a 6-well plate (0.5 × 10^6^ cells/well) one day prior to transfection. Transfection was performed on 70–80% confluent cells with 1.0 μg of empty pET01, wild-type (wt) and mutant (mut) minigenes using the Lipofectamine 2000 Reagent (Thermo Fisher Scientific) diluted in Opti-MEM Reduced Serum Medium according to the manufacturer’s instructions. All minigene transfection experiments were carried out in triplicate. The cells were harvested 24 h after transfection and total RNA was extracted using the Quick-RNA Miniprep Kit (Zymo Research, Irvine, CA, USA) with on-column DNase I digestion according to the manufacturer’s instructions. RNA concentration and quality were assessed using the Nanodrop One spectrophotometer (Thermo Fisher Scientific).

### 2.8. Reverse Transcription and RT-PCR Analysis

Five hundred nanograms of total RNA extracted from patient blood or minigene-transfected cells were reverse transcribed into cDNA using the SensiFAST cDNA Synthesis Kit (Bioline | Meridian Bioscience, London, UK (Bioline); Cincinnati, OH, USA (Meridian corporate)). RT-PCR was carried out using the GoTaq DNA Polymerase (Promega, Madison, WI, USA) with primers that span across exons 4–6 of the human *PPP1R12A* gene (PPP1R12A_EX4_FW and PPP1R12A_EX6_REV, Table S1) or vector-specific primers (pET01_EX1_RT-PCR_FW and pET01_EX2_RT-PCR_REV, Table S1). RT-PCR products were separated by electrophoresis on 2% agarose gel and visualized with Midori Green Advance (Nippon Genetics Europe, Düren, Germany) Original, uncropped gel images are provided in Figure S1. RT-PCR bands from minigene experiments were extracted from the gel using the NucleoSpin Gel and PCR Clean-up Kit (MACHEREY-NAGEL, Düren, Germany) and then bidirectionally sequenced with pET01_EX1_Seq_FW and pET01_EX2_Seq_REV primers (Table S1). The intensity of the bands was quantified with ImageJ Software, version 1.53k (NIH, Bethesda, MD, USA).

## 3. Results

### 3.1. Case Presentation

**Figure 1:**
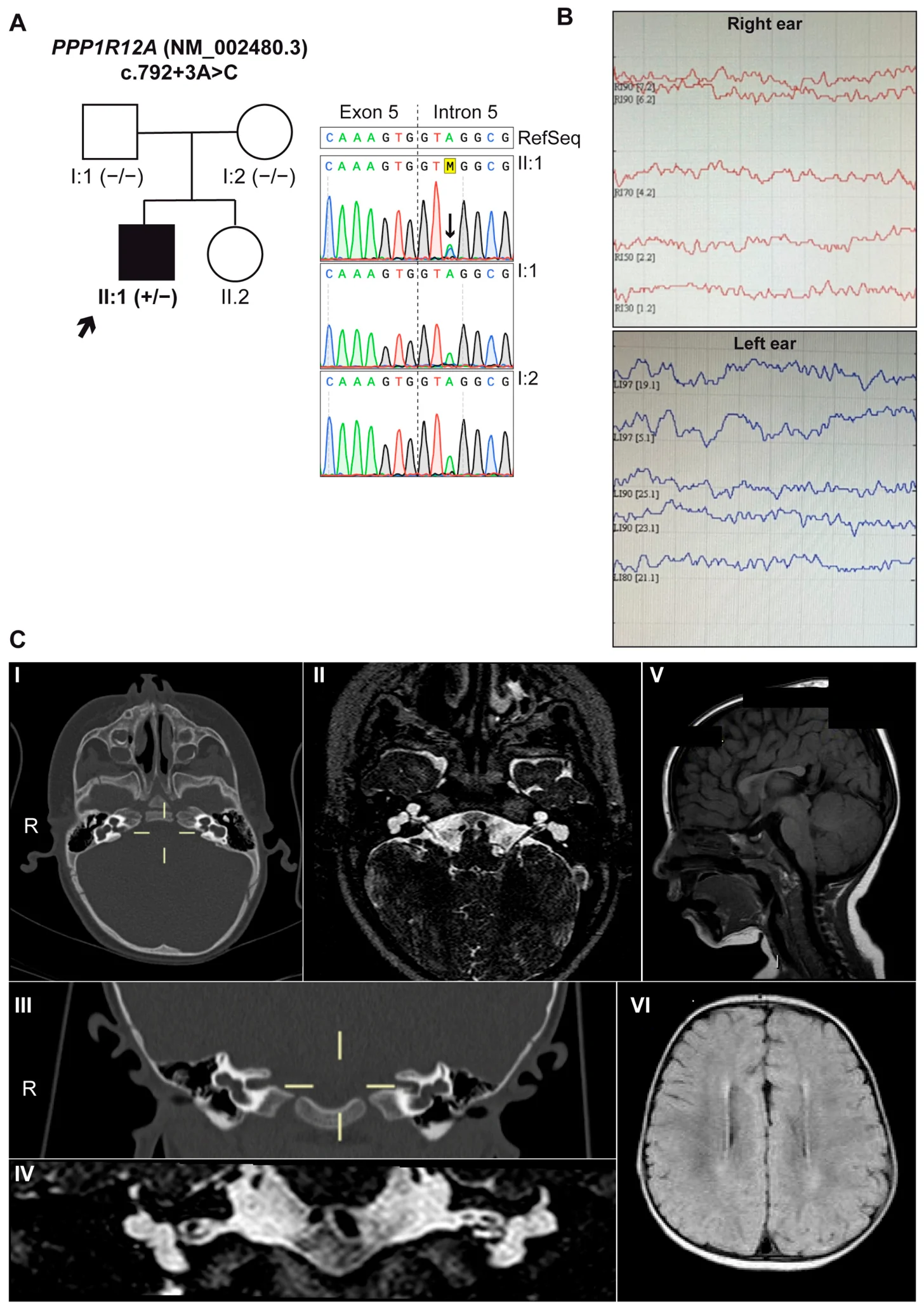
Pedigree and clinical data of the patient carrying a novel *PPP1R12A*-splicing variant. (**A**) Pedigree of the *PPP1R12A* patient family. The affected proband (II:1, black arrow) is indicated in black. *PPP1R12A* (NM_002480.3) c.792+3A>C variant genotyping is represented under the tested individuals in brackets (−/− homozygous wild-type; +/− heterozygous; II:2 not tested). Sanger sequencing electropherogram shows the heterozygous *PPP1R12A* c.792+3A>C variant in the proband. The unaffected parents are wild-type. The variant position is marked with a black arrow. (**B**) ABR testing in the proband, performed during natural sleep in a soundproof Faradized booth using an ICS Chartr system and inserted earphones for air-conduction and click stimulation, showed no waveforms bilaterally, consistent with absent responses. Red and blue traces correspond to the right and left ears, respectively. (**C**) HRCT scan of the ear in the axial (**I**) and coronal (**III**) planes and MRI study using T2w DRIVE sequence in the axial (**II**) and coronal (**IV**) planes show an inner ear malformation characterized by a globular appearance of the cochlea, with a single round chamber representing a common cavity configuration of the cochlea and vestibule. Associated aplasia of the lateral and posterior semicircular canals (SCCs) is observed, whereas the superior SCCs appear structurally preserved. Sagittal SE T1w brain MRI image (**V**) shows hypoplasia of the corpus callosum. Axial FLAIR image (**VI**) demonstrates hyperintensity of the deep white matter in the peri-trigonal regions, more pronounced on the left, consistent with delayed myelination in the terminal zones.

We report the case of a 12.5-year-old male patient, born from healthy unrelated parents, after an uncomplicated pregnancy and a eutocic delivery at 38 weeks of gestation (Figure 1A). At birth, his Apgar scores were 9 at 1 min and 10 at 5 min. Anthropometric measurements included a birth weight of 2.795 kg (25–50th percentile), a length of 47 cm (50th percentile), and a head circumference of 34 cm (50–75th percentile). Motor and cognitive developmental milestones were significantly delayed: the patient achieved unsupported sitting at 9 months, independent walking at 24 months and developed fluent speech around the age of 3 years. At the last clinical evaluation, anthropometric parameters were as follows: height 144.5 cm (25–50th percentile), weight 39 kg (25–50th percentile) and head circumference 55 cm (90–95th percentile). Dysmorphological examination revealed relative macrocephaly, frontal bossing, broad eyebrows and posteriorly rotated ears. Audiological evaluation displayed a bilateral, symmetric, profound SNHL. Investigations were performed following failure of the Newborn Hearing Screening Programme, which was conducted using transient-evoked otoacoustic emissions (TEOAEs). A diagnostic Auditory Brainstem Response (ABR) test was performed at three months of age to determine hearing thresholds. The test showed no reproducible waves (i.e., absent bilateral responses), a finding typically indicative of severe to profound hearing loss (Figure 1B). These results were later supported by behavioral audiological evaluation using free field audiometry test. After an initial trial with hearing aids, the patient underwent bilateral cochlear implant at 18 months of age, with good outcomes. Moreover, temporal bone high-resolution computed tomography (HRCT) revealed a complex bilateral cochlear malformation characterized by the presence of a single round chamber representing a common cavity configuration of the cochlea and vestibule, with associated aplasia of the lateral and posterior semicircular canals (SCCs). In contrast, the superior SCCs appeared structurally preserved (Figure 1C, panels I–IV). Clinical evaluation of the patient also included an ophthalmological examination, which identified a severe unilateral myopia in the right eye. In addition, renal ultrasound imaging displayed duplicated renal collecting system with right-sided megaureter and ureterocele. Transfontanellar cranial ultrasound showed areas of increased echogenicity in the periventricular white matter. Brain magnetic resonance imaging (MRI) demonstrated an underdeveloped corpus callosum and delayed white matter myelination (Figure 1C, panels V–VI). Finally, echocardiographic evaluation did not reveal any structural or functional cardiac abnormalities. The association of congenital profound bilateral SNHL with congenital malformations involving the inner ear, the genitourinary and the CNS was therefore consistent with a syndromic form of HL, supporting the need to investigate a potential genetic etiology (Table S2).

### 3.2. Molecular Analysis

First-tier genetic testing included Sanger sequencing of the *GJB2* gene and copy number variant analysis of *STRC* via multiplex ligation-dependent probe amplification (MLPA). Both tests were negative. Consequently, trio-based WES was performed and, given the patient’s hearing loss phenotype and the presence of an enlarged vestibular aqueduct (EVA), WES data were firstly analyzed focusing on known hearing loss-associated genes, excluding any recognized genetic cause. In addition, targeted evaluation of *SLC26A4* and other genes implicated in EVA pathogenesis (*FOXI1*, *KCNJ10*, *EPHA2*) [25] also revealed no pathogenic variants. Therefore, we proceeded to unfiltered WES data analysis, which led to the identification of a novel heterozygous non-canonical splice-site variant (c.792+3A>C), located in intron 5 of the *PPP1R12A* gene (NM_002480.3). The variant was absent in both parents, thus confirming its de novo origin (Figure 1A). According to the American College of Medical Genetics and Genomics and the Association for Molecular Pathology (ACMG/AMP) guidelines and SVI General Recommendations [21], as implemented in the VarSome variant interpretation platform (https://varsome.com/, accessed on 30 March 2026), the variant was classified as “likely pathogenic”. The classification was supported by the following criteria: (a) PM2_supporting (+1 point) due to the fact that it is absent from large population databases, including gnomAD; (b) PP3_strong (+4 points) since multiple calibrated in silico tools predicted a deleterious impact on splicing for this non-canonical splice-site variant [26]. Specifically, MaxEntScan predicted a reduction in splicing efficiency (score: 7.144), while scSNV-ADA (score: 0.999), scSNV-RF (score: 0.976), and SpliceAI (donor loss: 0.93; acceptor loss: 0.91) all indicated a high probability of splice site disruption; (c) PS2_strong (+4 points) considered that the variant was identified as de novo through trio-based WES and validated by Sanger sequencing, with its absence confirmed in both the mother and the father (maternity and paternity confirmed).

### 3.3. Splicing Minigene Assay and RT-PCR Analysis on Patient RNA

**Figure 2:**
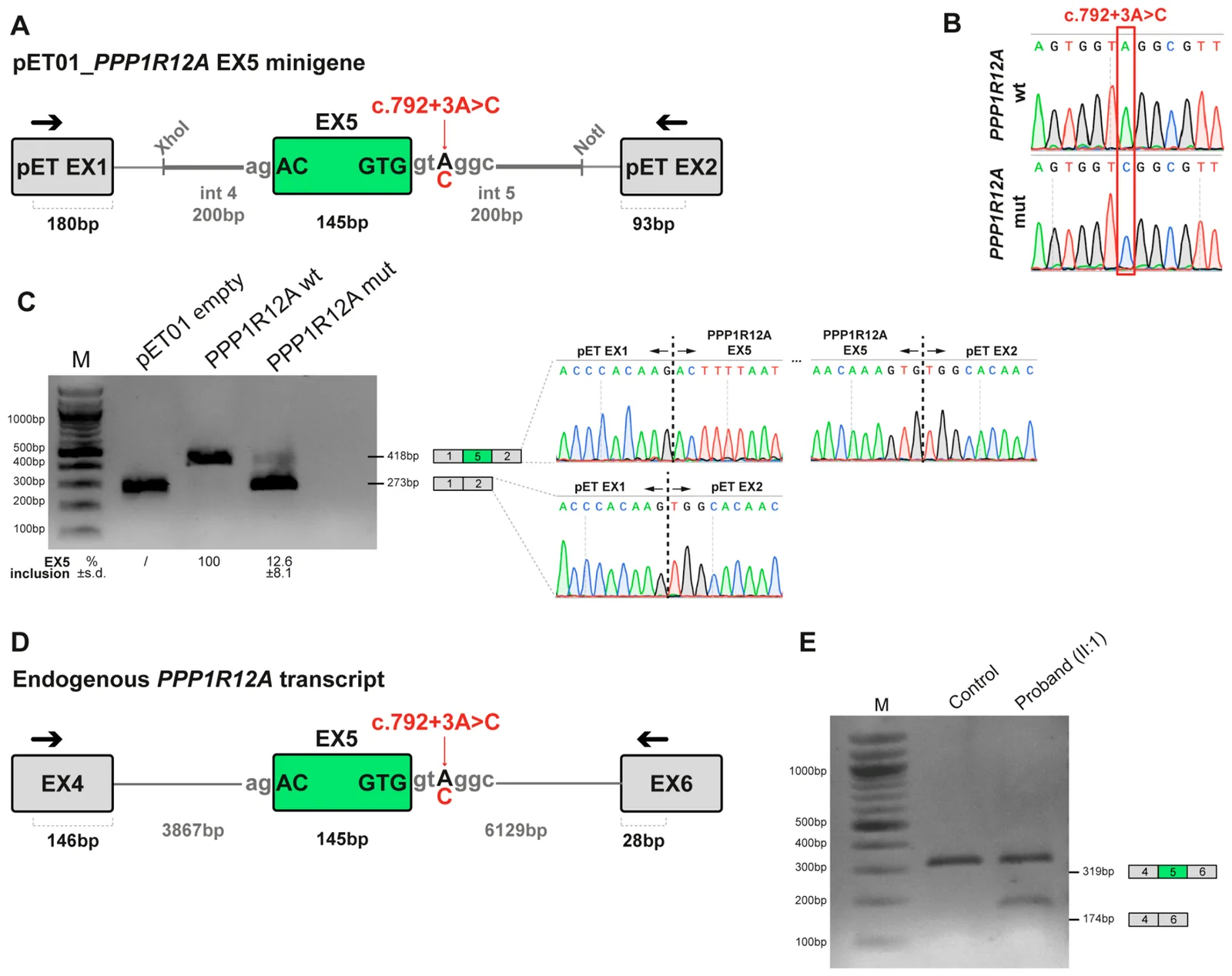
In vitro minigene splicing assay and RT-PCR analysis on the RNA from peripheral blood leukocytes of the patient. (**A**) Schematic representation of the minigene construct containing *PPP1R12A* exon 5 (EX5, green) with 200 bp of flanking intronic sequences (int4 and int5), cloned between the XhoI and NotI restriction sites of the pET01 exon-trap vector. Black arrows indicate primers located in pET01 exons 1 and 2, used for cDNA amplification. The sequences flanking the EX5–int5 junction in the wild-type (wt) and mutant (mut) minigenes are shown, with the c.792+3A>C substitution highlighted in red. (**B**) Sanger sequencing electropherograms confirming correct cloning of wt and mut minigenes and the presence of either the reference or the c.792+3A>C variant. (**C**) RT-PCR analysis using pET01_EX1_RT-PCR_FW and pET01_EX2_RT-PCR_REV primers of HEK293T cells transfected with empty pET01, wt or mut minigenes. Electrophoresis and sequencing of RT-PCR products revealed a single transcript for the wt construct and two transcripts for the mut construct. The difference between the two transcripts was due to the presence or absence of EX5, resulting in amplicons of 418 bp and 273 bp, respectively. Quantification (%) of EX5 inclusion was performed using ImageJ and is reported below the gel image as mean ± standard deviation (SD) of three independent experiments. (**D**) Schematic representation of the *PPP1R12A* genomic region from EX4 to EX6, with EX5 highlighted in green. (**E**) PCR amplification using PPP1R12A_EX4_FW and PPP1R12A_EX6_REV primers of cDNA from a healthy control and the proband (II:1) carrying the c.792+3A>C variant. The proband showed both the normally spliced transcript (319 bp) and an alternative transcript lacking EX5 (174 bp). M, 100 bp DNA Ladder (New England Biolabs).

To assess the impact of the c.792+3A>C variant on *PPP1R12A* mRNA splicing, a minigene assay was performed (Figure 2A,B). Analysis of RT-PCR products showed a full-length 418 bp transcript in cells transfected with the wild-type (wt) minigene, consistent with correct splicing. This size corresponds to the expected product comprising 273 bp of vector sequence and 145 bp of the target insert. Sanger sequencing of the 418 bp band confirmed accurate recognition of both the 3′ and 5′ splice sites of *PPP1R12A* exon 5 in the wt construct (Figure 2C). Conversely, transfection of cells with the mutant (mut) construct led to the production of two distinct transcripts: the correctly spliced transcript, identical in size to that produced by the wt construct (418 bp), represented the 12.6% ± 8.1 of the total transcripts, based on densitometric quantification of band intensities using ImageJ (mean ± SD of three independent experiments). The more abundant transcript was shorter in size (273 bp) and Sanger sequencing revealed that this smaller product resulted from complete skipping of *PPP1R12A* exon 5 (145 bp) (Figure 2C), indicating that the c.792+3A>C variant disrupts normal donor splice site recognition and induces aberrant mRNA splicing, generating an alternative transcript lacking exon 5.

The exon-skipped transcript (c.648_792del) introduced a frameshift and a premature termination codon (PTC), p.(Leu217Alafs*7) and is therefore predicted to undergo nonsense-mediated mRNA decay (NMD) [27,28], preventing the synthesis of a truncated, non-functional protein of 222 amino acids, instead of the full-length wild-type protein of 1030 amino acids (UniProtKB–O14974). However, NMD was not experimentally evaluated in the present study, and its involvement is therefore inferred from the predicted molecular consequence of the variant rather than directly demonstrated.

To confirm the splicing effect of the c.792+3A>C variant in vivo, RT-PCR was performed on RNA extracted from peripheral blood of the proband and a healthy control (Figure 2D,E). Agarose gel electrophoresis revealed the presence of two distinct transcripts in the proband. The first transcript corresponded to the canonical splicing isoform (319 bp). The second, shorter transcript of 174 bp, resulted from aberrant splicing due to complete skipping of exon 5. This exon-skipping event was consistent with the result observed in the minigene assay.

## 4. Discussion

In this study, we report a young male patient presenting with syndromic HL in which WES identified a novel heterozygous de novo splicing variant (c.792+3A>C), located in intron 5 of the *PPP1R12A* gene. Bioinformatic analysis predicted that this variant impairs the donor splice site at the 5′ end of the intron. Consistently, both the minigene splicing assay and in vivo RT-PCR analysis demonstrated that the c.792+3A>C variant causes the production of an aberrant mRNA transcript, with an insertion of a PTC within the fifth ankyrin repeat domain of the protein. Ankyrin repeat domains are highly conserved motifs involved in mediating protein–protein interactions and are essential for protein function. Altogether, these findings support a causal correlation between the novel splicing variant c.792+3A>C and the patient’s phenotype, thereby expanding the mutational spectrum of the *PPP1R12A* gene. In addition, the experimental evidence allows us to replace the PP3 criterion, which is based solely on bioinformatic predictions, with PVS1, despite the variant being located outside of the canonical splicing site (±1, 2). According to the ClinGen SVI Splicing Subgroup recommendations [26], the strength of PVS1 was reduced to PVS1_Strong, as the minigene assay demonstrated a near-complete proportion of alternative transcript compared with the wild-type transcript, supporting the application of PVS1 at a strong level of evidence. As shown in the Section 3, there was concordance between the minigene assay and patient RNA analysis in terms of whether or not a variant caused aberrant splicing. However, in the patient-derived RNA, transcripts originating from the unaffected wild-type allele mask the true relative proportions of the normal and aberrantly spliced transcripts produced by the mutant allele, preventing accurate quantification of the mutant allele-specific splicing outcome. In addition, the functional studies performed allowed us to apply also the PS3 criterion at moderate strength, and this updated assessment allowed us to reclassify the variant from likely pathogenic to pathogenic, with an increase in the ACMG evidence score from +9 to +11 points.

Previously, three different splicing variants were reported in the literature. Specifically, another splice site variant, also localized in intron 5 of *PPP1R12A*, the c.793-1G>A variant, has been reported in the HGMD Professional database as a disease-causing variant. This variant was identified as de novo in a 5.5-year-old young boy presenting with multiple congenital anomalies, including agenesis of the corpus callosum and renal asymmetry [8]. The c.793-1G>A variant is predicted to disrupt the canonical acceptor splice site of intron 5, leading to abnormal splicing. However, no functional validation has been reported to date. Another splicing variant, c.2666+3A>G in intron 20 of *PPP1R12A*, has been reported in the HGMD Professional database and described in a neonate with GUBS, who presented with multiple congenital anomalies but lacking typical central nervous system abnormalities [10]. Functional validation using a minigene assay confirmed the splicing-disruptive effect of the variant and supported a loss-of-function mechanism. The most recently reported splicing variant, c.1551-2A>G, localized in intron 11 of the *PPP1R12A* gene, was identified in twins presenting with GUBS and DSD, who exhibited phenotypic differences in genital development. According to ACMG/AMP criteria and given the canonical splicing localization, this variant has been classified as pathogenic [14] without functional validation.

To date, a total of 24 likely pathogenic or pathogenic variants in the *PPP1R12A* gene, including the novel variant here reported, have been described in association with GUBS and GUBS-related conditions (Table 1). These include nonsense (11/24, 45.8%), frameshift (7/24, 29.2%), splice site (4/24, 16.7%) and one stop-loss (1/24, 4.2%) variants, as well as one complex rearrangement (1/24, 4.2%). Most of these alterations are predicted to disrupt normal protein synthesis, leading to premature translational termination, NMD activation and likely resulting in haploinsufficiency, which is considered the primary pathogenic mechanism of the disease. In addition, Saul et al. (2026) reported a missense variant (c.38A>G, p.(Gln13Arg)) in a patient with short stature and jejunal atresia, in the absence of typical GUBS clinical signs [29]. However, neither the functional impact of this variant nor hypotheses regarding its potential pathogenic mechanisms, including haploinsufficiency, were discussed to support its pathogenic classification. Moreover, the reported variants are distributed throughout the entire gene and do not cluster in specific exons or protein domains. Therefore, no clear genotype–phenotype correlation has been established to date, as phenotypic variability does not appear to depend on the type or position of the variant. This highlights the need for comprehensive genetic analysis, functional studies, and detailed phenotypic characterization to better understand *PPP1R12A*-related disorders.

From a clinical perspective, our study represents the first documented case in which HL and inner ear malformation are observed as key clinical features in addition to the classical GUBS-associated phenotype. Specifically, consistent with the recurrent phenotype described in the *PPP1R12A*-related malformative syndrome, our patient shares with previously reported patients the core manifestations including genitourinary (duplicated renal collecting system and ureterocele) and CNS (hypoplasia of the corpus callosum and delayed myelination) malformations. This overlap confirms that the classical malformative phenotype is indeed present in our case and anchors it firmly within the known spectrum of the *PPP1R12A*-related condition. Beyond this shared core phenotype, our patient exhibits HL and inner ear malformation, which have not previously been fully characterized as part of the spectrum. In this context, Pandya and collaborators described a patient harboring a *PPP1R12A* nonsense variant with a complex phenotype, primarily characterized by epilepsy and holoprosencephaly, a severe congenital CNS malformation resulting from incomplete separation of the cerebral hemispheres. As additional clinical information, a history of bilateral SNHL was reported; however, no inner ear malformations were detected nor were audiometric data provided [17]. In contrast, HL in our patient is well documented and is associated with structural abnormalities of the inner ear. These two independent observations therefore broaden the known phenotypic spectrum associated with *PPP1R12A* variants and support the need to actively investigate hearing function in patients with GUBS. This consideration is particularly relevant in syndromic conditions, such as GUBS, or in individuals with neurodevelopmental disorders, in whom hearing loss may be difficult to recognize clinically unless specifically assessed through audiological evaluation. Placed alongside the other recently expanded features of the disorder (e.g., the association with possible DSD, gastrointestinal abnormalities and neurological symptoms), our findings reinforce the emerging view of *PPP1R12A*-related condition as a multisystem syndromic condition rather than a predominantly genitourinary malformative condition.

Although the role of MYPT1, encoded by *PPP1R12A*, in inner ear development has not yet been described, the patient’s phenotype suggests that early otic patterning was at least partially initiated, whereas subsequent morphogenesis was profoundly disrupted. Specifically, the presence of a basal cochlear turn argues against a complete failure of cochlear specification, while the absence of modiolar architecture and failure of further cochlear outgrowth point to defective structural remodeling of the developing cochlear duct. Likewise, the selective absence of the lateral and posterior semicircular canals suggests impaired morphogenesis of the vestibular labyrinth rather than a complete loss of vestibular identity. In addition, given the role of MYPT1 in cytoskeletal organization [8], alterations in its activity could disrupt the actomyosin-driven physical forces required for key morphogenetic processes, such as apical constriction and cellular elongation [30]. Mechanistically, MYPT1 functions as a regulatory subunit of myosin light chain phosphatase (MLCP), which counteracts the activity of myosin light chain kinase (MLCK) by promoting dephosphorylation of the myosin regulatory light chain [3]. Consistently, Yang et al. (2014) demonstrated that MLCK-deficient mice exhibit impaired hearing, supporting the hypothesis that tightly regulated actomyosin contractility is essential for normal auditory function [31]. Therefore, we hypothesize that a proper balance between MLCK-driven phosphorylation and MLCP-mediated dephosphorylation is required to maintain appropriate actomyosin dynamics in inner ear cells. Functional data from animal model add further complexity. Indeed, while complete *Mypt1* knockout is embryonic lethal, heterozygous mice show no phenotype [32]. This discrepancy among heterozygous murine model and patients with heterozygous *PPP1R12A* pathogenic variant may reflect species-specific differences in gene dosage sensitivity, compensatory mechanisms involving other PP1 regulatory subunits in mouse, or the limited phenotypic characterization in mouse models. Lastly, the limited number of patients and functional studies do not allow us to exclude different pathogenic mechanisms than haploinsufficiency. In this light, our study could pave the way for additional functional investigations, including in vitro models (i.e., inner ear organoids) to validate the key role of MYPT1 in inner ear morphogenesis and/or transgenic models carrying specific *PPP1R12A* variants, already proven to be disease-causing in humans, to better define underlying mechanisms.

In summary, we report the first Italian patient diagnosed with a *PPP1R12A*-related disorder presenting with hearing loss and inner ear malformation, expanding the clinical phenotype of this condition and highlighting the need to monitor hearing function in patients carrying pathogenic *PPP1R12A* variants. Moreover, we strongly emphasize the importance of functional studies to demonstrate and support the pathogenicity of novel variants, thereby improving our understanding of *PPP1R12A*-related disease mechanisms and refining genotype–phenotype correlations for this disorder.

## Figures and Tables

**Table 1:** *PPP1R12A* variants identified in GUBS and GUBS-related disorders.

#	Variant	Type (Consequence)	Protein Change	Location	Inheritance	Phenotype	dbscSNV Score	SpliceAI	Functional Studies	Ref.
1	c.223_224delAC	Del (fs)	p.Thr75Cysfs*8	Exon 1	de novo	Multiple congenital anomalies	/	/	/	[8]
2	c.466C>T	SNV (ns)	p.Gln156*	Exon 3	de novo	Holoprosencephaly and EEG anomalies	/	/	/	[17]
3	c.681dupT	Dup (ns)	p.Lys228*	Exon 5	de novo	Multiple congenital anomalies	/	/	/	[8]
**4**	**c.792+3A>C**	**SNV (5′ss)**	**/**	**Intron 5**	**de novo**	**Multiple congenital anomalies + HL**	**(0.999 | 0.976)**	**DL (0.93)**	**MSA;**	**This study**
									**Patient RNA**	
5	c.793-1G>A	SNV (3′ss)	/	Intron 5	de novo	Multiple congenital anomalies	(0.999 | 0.936)	AL (1.00)	/	[8]
6	c.960dupA	Dup (fs)	p.Glu321Argfs*6	Exon 8	de novo	Multiple congenital anomalies	/	/	/	[8]
7	c.1189delA	Del (fs)	p.Thr397Hisfs*42	Exon 9	de novo	Multiple congenital anomalies	/	/	/	[8]
8	c.1415C>G	SNV (ns)	p.Ser472*	Exon 10	unknown	Multiple congenital anomalies	/	/	/	[8]
9	c.1510C>T	SNV (ns)	p.Arg504*	Exon 11	de novo	Multiple congenital anomalies	/	/	/	[8]
10	c.1543G>T	SNV (ns)	p.Glu515*	Exon 11	de novo	GUBS	/	/	/	[9]
11	c.1551-2A>G	SNV (3′ss)	/	Intron 11	de novo	GUBS and DSD	(0.999 | 0.936)	AL (1.00)	/	[14]
12	c.1880delC	Del (fs)	p.Pro627Leufs*4	Exon 14	de novo	GUBS	/	/	/	[13]
13	c.2033_2034delCT	Del (ns)	p.Ser678*	Exon 15	de novo	Multiple congenital anomalies	/	/	/	[8]
14	c.2073dupA	Dup (fs)	p.Ser692Ilefs*2	Exon 15	unknown	Multiple congenital anomalies/GUBS	/	/	/	[8,9]
15	c.2253delA	Del (fs)	p.Asp752Metfs*36	Exon 16	unknown	Persistent Müllerian duct syndrome	/	/	/	[10]
16	c.2298C>A	SNV (ns)	p.Tyr766*	Exon 17	de novo	Persistent Müllerian duct syndrome	/	/	/	[10]
17	c.2533C>T	SNV (ns)	p.Arg845*	Exon 18	de novo	Epilepsy, infantile	/	/	WB; Co-IP	[11]
18	c.2573G>A	SNV (ns)	p.Trp858*	Exon 18	unknown	Multiple congenital anomalies	/	/	/	[8]
19	c.2666+3A>G	SNV (5′ss)	/	Intron 20	de novo	GUBS	(0.996 | 0.910)	DL (0.52)	MSA	[12]
20	c.2698C>T	SNV (ns)	p.Arg900*	Exon 21	de novo	Multiple congenital anomalies	/	/	/	[8]
21	c.2739_2740delCT	Del (fs)	p.Leu914Argfs*14	Exon 21	de novo	Multiple congenital anomalies	/	/	/	[8]
22	c.2741T>A	SNV (ns)	p.Leu914*	Exon 21	unknown	Persistent Müllerian duct syndrome	/	/	/	[10]
23	c.3092A>T	SNV (sl)	p.(*1031Leuext*71)	Exon 25	de novo	Intestinal atresia/DSD	/	/	/	[15,16]
24	CR: tandem triplication of 527 bp and 236 bp in EX 1			Exon 1	unknown	Persistent Müllerian duct syndrome	/	/	/	[10]

Summary of 24 likely pathogenic or pathogenic variants reported in the *PPP1R12A* gene in association with GUBS and related congenital anomalies, including the variant identified in this study (highlighted in bold). Variants are categorized by type, including single-nucleotide variants (SNVs), deletions (del), duplications (dup), and a complex rearrangement (CR). For each variant, the table reports the nucleotide change (cDNA level), variant type and consequence (fs, frameshift; ns, nonsense; 3′ or 5′ss, splice site variant; sl, stop-loss), protein effect, location within the gene, inheritance pattern, associated phenotype, in silico pathogenicity predictions for splicing variants calculated through the dbscSNV (AdaBoost (ADA) | Random Forest (RF) algorithms) and the SpliceAI (AL, acceptor loss; DL, donor loss) prediction tools, functional assays performed to investigate the effect of variant (MSA, minigene splicing assay; patient RNA analysis (RT-PCR); WB, Western blot; Co-IP, co-immunoprecipitation) and references to the original publications. The reference sequence for the *PPP1R12A* gene is NM_002480.3.

## Data Availability

The datasets generated and analyzed during the current study are not publicly available because of privacy and ethical restrictions, but are available from the corresponding author on reasonable request.

## Supplementary Materials

The following supporting information can be downloaded at https://www.mdpi.com/article/10.3390/genes17080856/s1, Table S1: List of primers used in the study. Table S2: Human Phenotype Ontology (HPO) IDs describing the clinical features of the proband. Figure S1: Original, uncropped and unadjusted images supporting all gels reported in the main article.

## Abbreviations

The following abbreviations are used in this manuscript:

GUBS	genitourinary and/or brain malformation syndrome
WES	whole-exome sequencing
PPP1R12A	protein phosphatase 1 regulatory subunit 12A
MYPT1	myosin light chain phosphatase target subunit 1
PP1c	protein phosphatase 1
HGMD	human gene mutation database
DSD	disorders of sex development
SNHL	sensorineural hearing loss
CNS	central nervous system
gDNAs	genomic DNAs
HEK293T	human embryonic kidney 293T cells
TEOAEs	transient-evoked otoacoustic emissions
ABR	auditory brainstem response
HRCT	high-resolution computed tomography
SCCs	semicircular canals
MRI	magnetic resonance imaging
MLPA	multiplex ligation-dependent probe amplification
EVA	enlarged vestibular aqueduct
ACMG/AMP	American College of Medical Genetics and Genomics/Association for Molecular Pathology
PTC	premature termination codon
NMD	nonsense-mediated mRNA decay
WT	wild-type
MUT	mutant
ADA	AdaBoost
RF	Random Forest
MLCP	myosin light chain phosphatase
MLCK	myosin light chain kinase
