# Supplementary material for: Phenotypic Expansion of PPP1R12A-Related Syndrome: A Novel Splicing Variant Associated with Hearing Loss and Inner Ear Malformations
Source: Genes (Basel). 2026 Jul 24;17(8):856. doi: 10.3390/genes17080856 (PMC13512303; doi:10.3390/genes17080856)
Supplement: Supplementary file 1 [file genes-17-00856-s001.zip › genes-4403108-supplementary.pdf]

Supplementary File

Primer	Sequence 5’ - 3'
PPP1R12A_XhoI_FW	TACTCGAGTGACTCAAGATCCTCTTTTTCTGAGTT
PPP1R12A_NotI_REV	TAGCGGCCGCAGAACCACCTCATACGGTAGC
pET01_EX1_RT-PCR_FW	GATCGATCCGCTTCCTGCCCC
pET01_EX2_RT-PCR _REV	CTGCCGGGCCACCTCCAGTGCC
pET01_EX1_Seq_FW	GGATTCTTCTACACACCC
pET01_EX2_Seq_REV	GCAGCACTGATCCACGATG
PPP1R12A_EX4_FW	GCAGCTCGAAAGGAAGAAGA
PPP1R12A_EX6_REV	CATCTGCTACATCAAAGGCTGT

Table S1. List of primers used in the study. Cloning primers contain the XhoI and NotI restriction sites (underlined) to subclone the amplified fragments in the XhoI/NotI pET01-digested vector. Primers for RT-PCR analysis of both endogenous and minigene-derived transcripts and primers used for sequencing are also reported.

Clinical feature	HPO IDs
Bilateral sensorineural hearing loss	HP:0008619
Abnormal inner ear morphology	HP:0011390
Aplasia of the semicircular canal	HP:0011381
Duplicated collectig system	HP:0000081
Congenital megaureter	HP:0008676
Ureterocele	HP:0000070
Abnormal periventricular white matter morphology	HP:0002518
Delayed CNS myelination	HP:0002188
Hypoplasia of the corpus callosum	HP:0002079
Myopia	HP:0000545
Motor delay	HP:0001270
Delayed speech and language development	HP:0000750
Relative macrocephaly	HP:0004482
Frontal bossing	HP:0002007
Broad eyebrow	HP:0011229
Posteriorly rotated ears	HP:0000358

Table S2. Human Phenotype Ontology (HPO) IDs describing the clinical features of the proband.

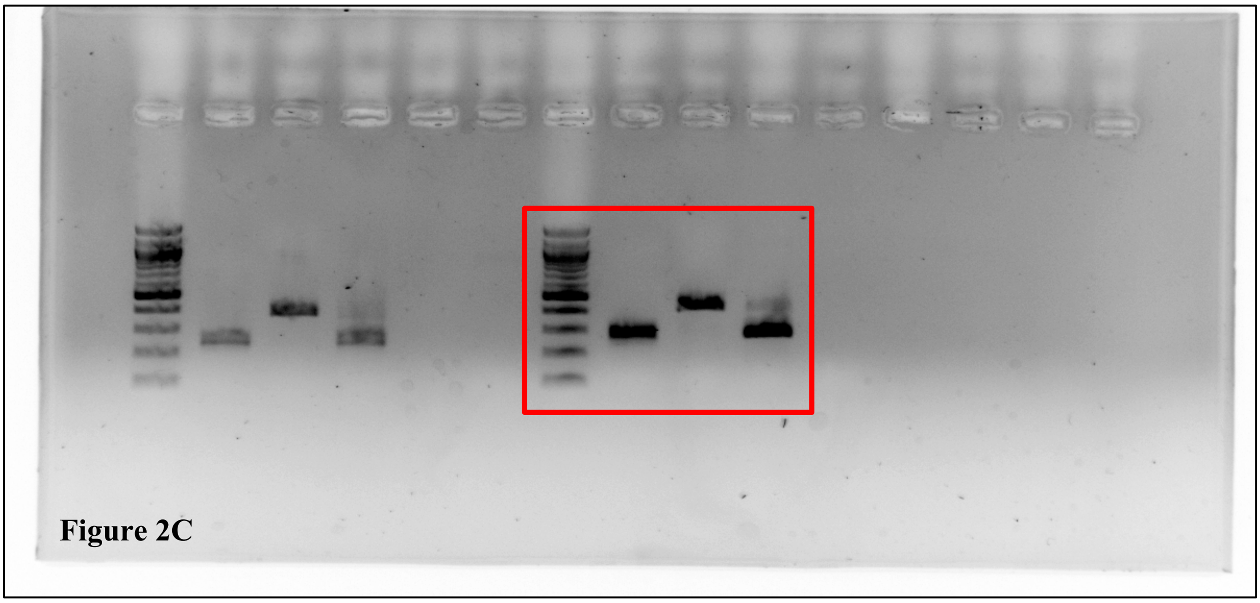

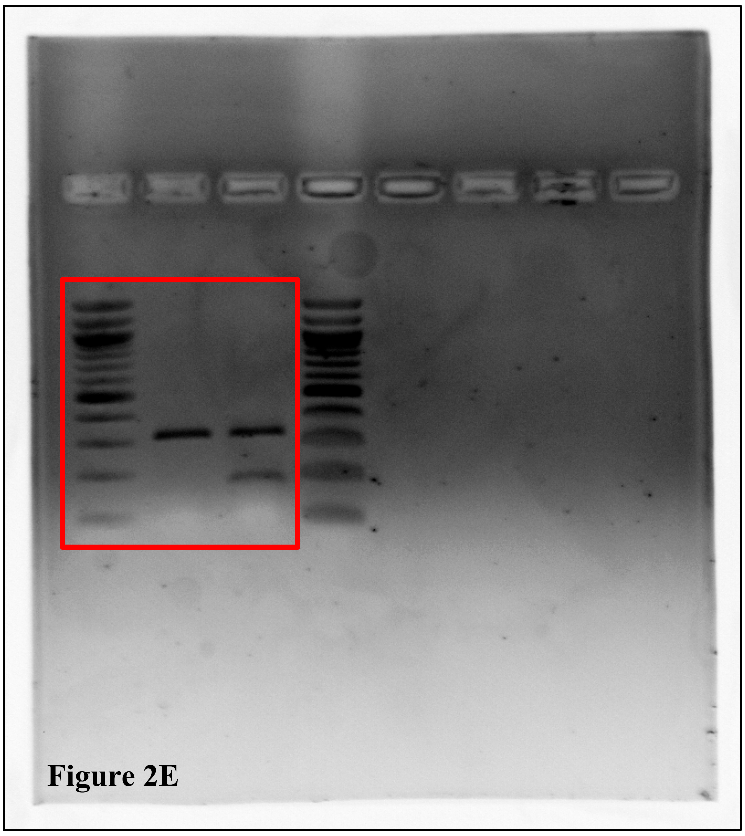

Figure S1. Original, uncropped and unadjusted images supporting all gels reported in the main article.
